# Legume-rhizobium specificity effect on nodulation, biomass production and partitioning of faba bean (*Vicia faba* L.)

**DOI:** 10.1038/s41598-021-83235-8

**Published:** 2021-02-11

**Authors:** Bayou Bunkura Allito, Nana Ewusi-Mensah, Vincent Logah, Demelash Kefale Hunegnaw

**Affiliations:** 1 Department of Plant and Horticultural Science, Hawassa University College of Agriculture, Hawassa, Ethiopia; 2 Department of Crop and Soil Sciences, Kwame Nkrumah University of Science and Technology, Kumasi, Ghana

**Keywords:** Rhizobial symbiosis, Ecosystem ecology

## Abstract

Greenhouse and multi-location experiments were conducted for two consecutive years to investigate the effects of rhizobium on nodulation, biomass production and partitioning of faba bean. Split-plot in randomized complete block design was used for field experiments. Treatments consisted of six rhizobium strains and three faba bean varieties. Peat carrier-based inoculant of each strain was applied at the rate of 10 g kg^−1^ seed. Non-inoculated plants without N fertilizer and with N fertilizer served as –N and + N controls, respectively. Data on nodulation, shoot dry weight and root dry weight were collected and analyzed. Inoculation of rhizobium significantly increased nodulation of faba bean under greenhouse and field conditions. Location x strain x variety interaction had significant effects on nodulation, dry matter production and partitioning. Rhizobium inoculation increased nodulation, shoot and root dry weights of faba bean across locations. For example, inoculation with rhizobium strains NSFBR-15 and NSFBR-12 to variety Moti resulted in 206.9 and 99.3% shoot dry weight increase at Abala Gase and Hankomolicha, respectively and 133.3 and 70.7% root dry weight increase on the same variety at the same sites, respectively. Nodulation and biomass production depend on the compatibility between faba bean genotype and rhizobium strain and its interaction with soil bio-physical conditions.

## Introduction

Nitrogen fixation in legume-rhizobium symbiosis depends on the genotype of legume, rhizobium strain and the interactions of these with the bio-physical environment^1^. Each legume species requires specific rhizobium strain for effective nodulation and nitrogen fixation^2^. The amount of nitrogen fixed therefore varies with legume species and/or variety^3^ and effectiveness of partner microsymbiont^4^. Keneni et al.^5^ revealed that both legume cultivar and rhizobium strain can affect nodulation and nitrogen fixation. Ouma et al.^6^ showed that, host-specific rhizobia strains of common bean and soybean adapted better to the local soil and environmental condition. Better survival rate and soil persistence in turn increase the chance of successful nodulation and nitrogen fixation^7^.

Faba bean rhizobia are diverse at species, plant growth promoting ability and symbiosis related gene levels^8^. The crop usually forms effective nitrogen fixing symbiosis with strains of *Rhizobium leguminosarum* symbiovar viciae (Rlv)^9^ which are also diverse and ubiquitous^4,10^. Sanchez-Canizares et al.^11^ demonstrated the wide diversity of plasmids within Rlv species, especially for the symbiotic plasmid. Therefore, roots of growing plant are exposed to various Rlv strains’ populations^12,13^. Nodulation of plant with ineffective and inefficient strains are likely to occur as natural Rlv populations in the soil are quantitatively and qualitatively heterogeneous^14^. This point out that optimum nodulation and nitrogen fixation requires inoculation with host-specific effective rhizobium strains.

In this regard, inoculation with effective and appropriate rhizobial strain is necessary to improve symbiotic nitrogen fixation and optimize faba bean productivity^15^. Inoculation affects microbial community by increasing desired rhizobia strain population in the rhizosphere^16^. For successful establishment, inoculants strain must be able to survive in soil environment and take advantage of ecological niche to be offered by the roots of host plant^17^. Otherwise, poorly efficient Rlv strains may outcompete and gain advantage over effective rhizobium strains used for inoculation^18^. Since the soil may harbor certain ineffective nodule forming native rhizobia, effective nodule formation largely depends upon the competiveness of inoculants strain. This upholds that strain competiveness is key for successful inoculation under field conditions^19^. Therefore, symbiotic performance depends on the abundance of effective rhizobia strain and its competiveness for nodulation^12^.

It is evident that, there are diversified faba bean cultivars in sub-Saharan Africa which are likely to be accompanied by symbiotically effective nitrogen fixing indigenous rhizobium strains. Researches to date have mainly focused on isolation and screening of native rhizobia for symbiotic effectiveness under greenhouse conditions. There is therefore a research gap in determining rhizobium strains and faba bean cultivars interaction effects to enhance nodulation and N-fixation. Inferences based on research results in laboratory and under greenhouse conditions do not always reflect the reality of rhizobium strain performance under field conditions^20^. Thus, the aim of this research was to evaluate the performance of some selected rhizobium strains on nodulation and biomass yield of improved faba bean varieties under different agro-ecological zones of southern Ethiopia.

## Materials and methods

### Description of field experimental locations

Four different locations were selected in two major faba bean growing agro-ecologies (cool-humid and cool sub-humid) in southern Ethiopia. Two locations, namely Hankomolicha and Abala Gase in cool humid and two locations, namely Haranfama and Gike Atoye in cool sub-humid agro-ecological zones were selected for field experiments. The experimental locations in cool humid and cool sub-humid agro-ecological zones received 1472.9 mm and 1092.5 mm mean annual rainfall (Table 1), respectively. The distribution of rainfall in both agro-ecologies is bimodal. Minor rainy season occurs from February to April whereas the major rainy season spans from June to September. In each agro-ecology, two experiments were conducted at selected locations during the major rainy season of 2016 and 2017.

**Table 1:** Annual average rainfall, mean maximum and minimum temperatures during the study period.

Year		Cool humid (location: HK and AG)			Cool sub–humid (location: HR and GA)		
		Rain fall	^a^Max	^b^Min	Rain fall	^a^Max	^b^Min
		mm	°C	°C	mm	°C	°C
2016	June	179.6	14.1	7.7	127.6	21.3	12.9
	July	133.5	16.4	5.6	96.8	24.3	12.5
	August	182.2	16.3	6.1	191.5	22.8	11.6
	September	159.6	16.5	7.2	104	23.7	13.4
	Annual	1477.6	17.1	8.1	1303	25.2	15.1
2017	June	62.8	17.0	9.2	35.0	25.1	15.3
	July	218.8	15.6	5.2	160.7	23.3	11.9
	August	218.8	14.1	6.5	165.7	20.9	11.3
	September	206.0	14.0	7.8	204.1	19.1	10.9
	Annual	1590.6	17.4	9.3	1199.2	24.5	14.4
Ten years’ annual average (2008–2017)		1472.9	15.4	7.1	1092.5	22.4	11.7

^a^*Max* Maximum temperature.

^b^*Min* Minimum temperature, *HM* Hankomolicha, *AG* Abala Gase, *HR* Haranfama, *GA* Gike Atoye.

### Soil sampling and analyses

Pre-sowing soil samples were collected from each location. Samples were cored to a depth of 20 cm from 20 random spots from each entire experimental area to form composite sample. Soil chemical and physical properties were then analyzed (Table 2). Determination of soil particle size distribution was carried out by the Bouyoucos hydrometer method^21^. Bulk density was determined using the core sampling method as described by Black and Hartge^22^. The soil pH was determined in 1:2 soil to water solution ratio using a pH meter as described by Carter and Gregorish^23^. Exchangeable acidity was determined by extracting the soil samples with 1.0 M KCl solution and titrating against sodium hydroxide as described by McLean^24^.

**Table 2:** Physical and chemical properties of surface soils (0–20 cm) and rhizobia populations at the studylocations.

Soil parameters	Study locations			
	Hankomolicha	Abala Gase	Haramfama	Gike Atoye
pH (1:2; Soil:H_2_O)	6.57	5.37	6.02	5.60
Avai. P (mg kg^−1^)	12.60	5.70	8.40	6.03
Total nitrogen (%)	0.17	0.17	0.16	0.22
Organic carbon (%)	2.06	2.22	1.75	2.34
Organic matter (%)	3.55	3.83	3.02	4.03
C:N ratio	12.10	13.10	11.30	10.80
CEC (meq/100 g)	29.40	27.56	22.60	32.81
Exchangeable bases cmol_(+)_ Kg^−1^				
Na	2.11	0.93	0.95	0.83
K	3.14	0.75	2.36	1.25
Ca	13.40	15.09	12.60	17.73
Mg	7.22	5.38	6.44	5.20
Exc. acidity (cmol_(+)_ kg^−1^)	0.40	0.48	0.12	0.52
Bulk density (g/cm^3^)	1.24	1.21	1.35	1.25
Textural class	Clay	Clay loam	Loam	Clay
Rhizobial density (g^−1^ soil)	5.8 × 10^2^	1.0 × 10^3^	3.1 × 10^2^	1.7 × 10^3^

Exchangeable bases and cation exchange capacity (CEC) of the soils were determined by the 1.0 M ammonium acetate (pH 7) method, whereas exchangeable Ca, Mg, Na and K in the extract were measured by atomic absorption spectrophotometer (AAS)^25,26^. Organic carbon was determined following wet oxidation methods^27^. Total nitrogen of the soils was determined through digestion, distillation and titration procedures of macro-Kjeldahl method as described by Bremner and Mulvaney^28^. Available phosphorus in the soil was determined using sodium bicarbonate extraction solution (pH 8.5) method and the amount was measured by atomic absorption spectrophotometer as described by Olsen et al.^29^.

### Estimation of soil rhizobia population

The populations of native rhizobia, which could nodulate faba bean was determined by plant infection technique^30^ using modified Leonard jar assembly. A most-probable number (MPN) count was used to determine the number of viable and infective rhizobia at each site. Plastic pots assembly consisted of a 300 ml capacity pot was filled with well-washed river sand and moistened by adding 200 ml of the N-free nutrient solution. Each pot assembly was wrapped with aluminum foil and autoclaved for 2.0 h at 121 °C. Serial tenfold dilution was prepared from each soil sample obtained from experimental sites. A 10 g of soil was diluted in 90 ml sterilized distilled water and shaken vigorously to make 10^–1^ dilution. Series of dilutions were then made from 10^–1^ to finally achieve 10^–10^, and subsequently used to inoculate faba bean seedlings grown on sterilized sand using four replications. Distilled water alternated with N free nutrient solution was used to water the plants. Nodule observations were made after 45 days of inoculation. The enumeration of native rhizobia most-probable-number (MPN) method^31^ revealed that the mean populations of native faba bean nodulating rhizobia at the experimental sites (Table 2) varied from 3.1 × 10^2^ to 1.7 × 10^3^ cells per gram of soil. The highest soil native rhizobial population was observed at Gike Atoye followed by at Abala Gase experimental site.

### Treatments and experimental design

The study included both greenhouse and field experiments. Six rhizobial strains (NSFBR-12, NSFBR-15, NSFBR-20, HUFBR-17, TAL_1035 and EAL-110), originally collected by Haremaya University, Holleta Agricultural Research Center and National Soil Laboratory (NSL) in Ethiopia were used for the study. The inoculum was used at the concentration of approximately 10^9^ cells g^−1^ in peat carrier. Purity of strain cultures was assessed in the Soil Microbiology Laboratory at Holleta Agricultural Research and Haremaya University. Sterility of the carrier was checked before mixing with the rhizobial culture. Seeds of three nationally registered faba bean varieties (Dosha (COLL 155/00-3), Moti (EH 95078-6), Gora (EKOl024-1-2) were collected from Holleta Agricultural Research Centers and used for the study.

### Greenhouse experiment

Treatments comprised a factorial combination of six rhizobium strains (NSFBR-12, NSFBR-15, NSFBR-20, HUFBR-17, TAL_1035 and EAL-110) and three faba bean varieties (Moti, Dosha and Gora). Three replicate modified Leonard jars filled with washed and sterilized river-sand were used for each treatment (strain × variety) arranged in a completely randomized design (CRD). Seed inoculation was done just before planting.

Uniform seeds of good viability were surface sterilized with ethanol and hydrogen peroxide^32^ before applying carrier-based inoculants. Two healthy seeds inoculated with the required stain were sown per pot at 4 cm depth and later thinned to one after seedling emergence. Parts of non-inoculated seedlings grown in jars were supplied with nitrogen while others were grown without nitrogen as positive (+ N) and negative (−N) control treatments, respectively^32^. Both inoculated and non-inoculted –N control plants were supplied with nitrogen-free nutrient solution^33^. Plants were grown in a greenhouse with average day and night temperature of 28 °C and 18 °C, respectively and harvested 60 days after planting^34,35^.

The experimental pots were excessively irrigated at harvest to uproot the plants with minimum disturbance of intact nodules. The roots were then carefully washed to remove sand particles after which nodules were removed and counted. Nodules from each plant were put in pre-labeled separate paper envelope and oven dry at 70 °C for 48 h, and weighted on sensitive balance.

### Field experiments

A split-plot in a randomized complete block design (RCBD) was used with four replicates nested at four different locations. Main plot treatments consisted of six rhizobium strains (NSFBR-12, NSFBR-15, NSFBR-20, HUFBR-17, TAL_1035 and EAL-110). Non-inoculated plants supplied with and without N fertilizer served as + N and -N controls, respectively. Sub-plot treatments were three faba bean varieties (Moti, Dosha and Gora).

Land preparation was done following conventional practices to make the field suitable for planting and divided into blocks and further into individual plots. Plot sizes measured 4 × 4 m (16 m^2^). Each variety was planted in 10 rows plot of 4 m length per main plot. The inter-row and intra-row spacing was maintained at 40 cm and 10 cm, respectively. Spacing between sub-plots and main plots were 1 m and 1.5 m, respectively. Peat carrier-based inoculant of each strain was applied at the rate of 10 g kg^−1^ seed^36^. A 1.0 kg seeds applied with 10 g inoculant were suspended in 1:1 ratio in 10% sugar solution in order to ensure that all the applied inoculum stuck to the seed. The thick slurry of the inoculant was gently mixed with dry seed so that all seeds received a thin coating of the inoculant. Inoculation was done just before planting under shade to maintain the viability of rhizobial cells.

The seeds were sown at a depth of about 4 cm. Phosphorus was applied to all plots in the form of triple-superphosphate (TSP) at the recommended rate of 46 kg P_2_O_5_ ha^−1^ at planting. Nitrogen fertilizer was applied two times in equal split doses to non-inoculated + N control treatment, at planting and six weeks after sowing at a recommended rate of 46 kg N ha^−1^. All other crop management and protection practices were applied uniformly to plots.

### Data collection and analyses

Shoot and root dry weights were measured at late-flowering stage (after 60 days of planting) and used to estimate total dry matter. Six plants from the middle rows of each plot were randomly selected and uprooted with ball of surrounding soils using a spade so as to obtain intact roots and nodules. Shoots were then cut from the roots at the collar, and partitioned into root and shoot. The adhering soil on the roots was carefully removed, and the roots with intact nodules were washed gently using tap water on a metal mesh sieve.

The nodules on the roots were picked and counted. Total sum of the nodules from six sampled plants plot^−1^ was averaged to obtain number of nodules plant^−1^. The nodules of six plants sampled from each plot were put together and immersed in a 50 ml capacity plastic cylinder which was filled to 30 ml with water. The volume of displaced water was recorded and the average value recorded as volume of nodules plant^−1^. All nodules from the six plant plot^−1^ were pooled and oven dried at 70 °C for 48 h and the average nodule weight plant^−1^ was estimated. Root and shoot samples were further cut into small pieces and placed in separate labeled paper bags. All samples were oven-dried at 70 °C for 48 h to determine average dry weight plant^−1^ in each plot. Shoot to root ratio was determined and used for root biomass estimation at later stages^37^.

The data were subjected to Analysis of Variance (ANOVA) using SAS^38^ computer software (SAS Institute Inc.). Combined analysis of variance was done to test significance between locations, rhizobium strains, faba bean varieties and interactions among these three factors for all measured parameters. Mean separations and comparisons were done using PROC GLM. Pearson correlation test was conducted to determine the associations among treatment means at p ≤ 0.05 probability level.

## Results

### Nodulation in greenhouse

All of the rhizobium strains evaluated in the greenhouse induced nodules on the faba bean varieties (Table 3). Nodulation was however not observed in non-inoculated (−N and + N) control treatments. Rhizobium strains NSFBR-15, TAL_1035, NSFBR-12 EAL-110 and NSFBR-20 produced significantly higher number of nodules plant^−1^ relative to HUFBR-17. There was a significant (p ≤ 0.05) strain x variety interaction effect for nodulation (Table 3). Strain NSFBR-15 when inoculated on the most prolific varieties Dosha and Gora induced 50.3 and 45.3 nodules plant^−1^, respectively. The strain NSFBR-15 also produced the highest nodule dry weight plant^−1^ (227.8 g) followed by NSFBR-12 which resulted in 210.6 g nodule dry weight plant^−1^. Rhizobium strain HUFBR-17 produced the fewest number of nodules and nodule dry weight plant^−1^ among the tested strains.

**Table 3:** Rhizobium strain x faba bean variety interaction effects on nodule numbers (NN) and nodule dry weight (NDW) per plant under greenhouse condition.

Parameters		Rhizobium strains										
NN	Varieties	TAL_1035		NSFBR-15		HUFBR-17		NSFBR-12		EAL-110		NSFBR-20
	Moti	34.3^b^		34.3^a^		31.7^a^		46.7^a^		40.3^ab^		36.0^ab^
	Dosha	37.3^b^		50.3^a^		27.7^a^		38.7^a^		33.0^b^		43.3^a^
	Gora	58.0^a^		45.3^a^		22.7^a^		38.3^a^		47.0^a^		31.7^b^
	Mean	43.2		43.3		27.3		41.2		40.1		37.0
	*CV (%)*	**11.3**										
NDW	Moti	167.0^a^		178.7^b^		149.0^a^		235.3^a^		197.7^ab^		176.3^ab^
	Dosha	188.7^a^		262.7^a^		130.0^a^		198.0^a^		161.0^b^		210.0^a^
	Gora	202.3^a^		242.0^a^		105.3^a^		198.3^a^		222.3^a^		148.7^b^
	Mean	186.0		227.8		128.1		210.6		193.7		178.3
	*CV (%)*	**11.2**										

Mean values in the same column with different letter(s) are significantly different at p ≤ 0.05 probability level.

### Nodulation under field condition

Rhizobium strains inoculation resulted in significant (p ≤ 0.05) increase in nodule number, nodule dry weight and nodule volume plant^−1^ relative to non-inoculated controls (Tables 4, 5, 6). Location x strain x variety interaction had significant (p ≤ 0.01) effect on number of nodules plant^−1^. Non-inoculated –N and + N control treatments resulted in the lowest number of nodules plant^−1^. Variety Dosha inoculated with NSFBR-15 produced the highest number of nodules plant^−1^ (88.5) followed by variety Gora inoculated with TAL_1035 and variety Moti inoculated with NSFBR-12 which produced 80.7 and 70.6 nodules plant^−1^ respectively at Abala Gase (Table 4).

**Table 4:** Rhizobium strain × faba bean variety interaction effects on nodule number at the study locations.

Rhizobium strain	Hankomolicha			Haranfama			Abala Gase			Gike Atoye		
	Moti	Dosha	Gora	Moti	Dosha	Gora	Moti	Dosha	Gora	Moti	Dosha	Gora
	(NN plant^−1^)			(NN plant^−1^)			(NN plant^−1^)			(NN plant^−1^)		
TAL_1035	49.9^b^	52.5^a^	58.3^a^	41.3^a^	41.7^a^	49.9^a^	50.2^b^	54.5^c^	80.7^a^	40.8^c^	42.6^b^	69.9^a^
NSFBR-15	54.0^a^	50.0^a^	45.7^b^	41.2^a^	43.5^a^	45.9^b^	39.3^ cd^	88.5^a^	53.6^b^	49.8^ab^	33.3^c^	65.4^a^
HUFBR-17	47.1^b^	50.2^a^	43.6^bc^	40.7^a^	34.4^b^	35.2^d^	36.1^de^	33.4^e^	32.5^d^	48.2^ab^	39.4^b^	25.3^e^
NSFBR-12	46.7^b^	44.3^b^	47.4^b^	40.2^a^	37.7^b^	42.0^c^	70.6^a^	48.6^c^	45.8^c^	53.8^a^	53.4^a^	54.6^b^
EAL-110	47.5^b^	35.9^bc^	40.7^ cd^	36.5^bc^	37.3^b^	35.1^d^	45.7^bc^	41.4^d^	53.9^b^	46.4^b^	30.1^c^	53.2^bc^
NSFBR-20	31.7^c^	35.3^c^	37.4^d^	38.7^ab^	34.3^b^	32.3^d^	42.6^ cd^	64.2^b^	42.8^c^	51.0^ab^	49.0^a^	40.6^d^
N^+^	22.7^e^	21.9^d^	23.1^e^	27.8^d^	30.5^c^	23.6f.	31.1^e^	27.9^e^	25.0^e^	33.3^d^	23.5^d^	48.0^c^
N^-^	26.8^d^	32.8^c^	36.2^d^	34.2^c^	37.7^b^	28.6^e^	22.4f.	27.8^e^	21.8^e^	31.6^d^	35.4^bc^	25.5^e^
*CV*_*a*_*(%)*		8.9			8.2			14.7			10.1	
*CV*_*b*_*(%)*		8.2			6.7			10.2			11.1	

Mean values in the same column with different letter(s) are significantly different at p ≤ 0.05 probability level; *NN* Nodule numbers.

**Table 5:** Rhizobium strain × faba bean variety interaction effects on nodule dry weight at the study locations.

Rhizobium strains	Hankomolicha			Haranfama			Abala Gase			Gike Atoye		
	Moti	Dosha	Gora	Moti	Dosha	Gora	Moti	Dosha	Gora	Moti	Dosha	Gora
	(mg plant^−1^)			(mg plant^−1^)			(mg plant^−1^)			(mg plant^−1^)		
TAL_1035	247.5^b^	260.3^a^	295.8^a^	211.3^a^	213.3^a^	261.3^a^	249.6^b^	271.8^bc^	414.3^a^	209.5^d^	219.2^b^	370.7^a^
NSFBR-15	275.0^a^	258.0^a^	239.0^b^	212.5^a^	226.5^a^	240.3^b^	203.5^c^	463.9^a^	286.7^b^	258.0^ab^	174.0^c^	346.9^a^
HUFBR-17	216.3^c^	235.8^b^	205.5^c^	198.5^ab^	163.3^ cd^	167.8^d^	163.5^d^	155.7^e^	151.0^e^	232.4^bcd^	184.0^c^	118.2^e^
NSFBR-12	228.8^bc^	224.5^b^	239.5^b^	207.3^ab^	192.0^b^	216.8^c^	346.7^a^	248.8^c^	234.3^ cd^	278.9^a^	272.7^a^	284.3^b^
EAL-110	236.0^bc^	174.8^c^	193.5^ cd^	176.0^ cd^	181.3^bc^	168.8^d^	228.3^bc^	201.3^d^	254.5^bc^	220.3^ cd^	144.1^de^	252.2^c^
NSFBR-20	155.5^d^	173.8^c^	177.8^d^	188.5^bc^	164.0^ cd^	153.0^de^	209.1^c^	317.3^b^	201.5^d^	245.7^bc^	230.7^b^	188.5^d^
+ N	112.3^e^	106.0^d^	111.5^e^	148.5^e^	156.8^d^	139.8^ef^	154.6^d^	135.1^e^	120.5^ef^	182.4^e^	121.9^e^	299.0^b^
-N	122.3^e^	154.5^c^	175.3^d^	162.8^de^	182.3^bc^	131.5f.	100.1^e^	129.3^e^	105.3f.	147.2f.	169.5^ cd^	113.9^e^
*CV*_*a*_*(%)*		7.7			9.5			15.6			10.0	
*CV*_*b*_*(%)*		8.7			7.6			10.7			11.4	

Mean values in the same column with different letter(s) are significantly different at p ≤ 0.05 probability level.

**Table 6:** Rhizobium strain x faba bean variety interaction effects on nodule volume at the study locations.

Rhizobium strains	Hankomolicha			Haranfama			Abala Gase			Gike Atoye		
	Moti	Dosha	Gora	Moti	Dosha	Gora	Moti	Dosha	Gora	Moti	Dosha	Gora
	(ml plant^−1^)			(ml plant^−1^)			(ml plant^−1^)			(ml plant^−1^)		
TAL_1035	2.9^ab^	3.1^a^	3.2^a^	2.4^a^	2.4^a^	2.7^a^	2.9^b^	3.1^c^	4.0^a^	2.4^abc^	2.4^bc^	3.5^a^
NSFBR-15	3.2^a^	2.9^ab^	2.7^b^	2.4^a^	2.5^a^	2.7^a^	2.4^bc^	4.7^a^	3.1^b^	2.8^a^	2.0^c^	3.7^a^
HUFBR-17	2.7^b^	2.8^ab^	2.5b^cd^	2.2^a^	1.9^bc^	1.9^bc^	2.1^ cd^	1.9^e^	2.0^ cd^	2.6^ab^	2.2^c^	1.5^d^
NSFBR-12	2.6^b^	2.5^bc^	2.6^bc^	2.1^ab^	2.1^abc^	2.3^ab^	3.6^a^	2.7^ cd^	2.5^bc^	2.7^a^	2.8^ab^	2.9^b^
EAL-110	2.8^ab^	2.1^ cd^	2.4b^cd^	2.1^ab^	2.2^ab^	2.0^bc^	2.6^bc^	2.5^d^	3.1^b^	2.6^ab^	1.9^ cd^	3.0^b^
NSFBR-20	1.8^c^	2.2^ cd^	2.2^ cd^	2.1^ab^	2.1^abc^	1.9^bc^	2.3^c^	3.8^b^	2.6^b^	2.7^a^	3.0^a^	2.4^c^
+ N	1.4^c^	1.3^e^	1.4^e^	1.7^b^	1.7^c^	1.4^d^	2.0^ cd^	1.8^e^	1.7^de^	2.1^bc^	1.5^d^	2.9^b^
-N	1.6^c^	2.0^d^	2.1^d^	2.0^ab^	2.3^ab^	1.6^ cd^	1.5^d^	1.9^e^	1.4^e^	2.0^c^	2.2^c^	1.6^d^
*CV*_*a*_*(%)*		19.0			19.5			24.3			19.2	
*CV*_*b*_*(%)*		9.9			10.5			10.2			13.4	

Mean values in the same column with different letter(s) are significantly different at p ≤ 0.05 probability level.

Table 5 shows increased nodule dry weight (NDW) plant^−1^ following inoculation relative to non-inoculated control treatments across the study locations. Inoculation with rhizobium strains significantly (P < 0.01) increased nodule dry weight compared to non-inoculated faba bean plants at all study locations. Analysis of variance across the study locations revealed that, location × rhizobium strain × variety (L × R × V) interaction had significant effect on nodule dry weight plant^−1^. Inoculation of NSFBR-15 to variety Dosha and TAL_1035 to variety Gora resulted in the first (463.9 mg/plant) and the second (414.3 mg/plant) highest nodule dry weight plant^−1^, respectively at Abala Gase (Table 5). Variety Gora inoculated with strain TAL_1035 produced the highest nodule dry weight plant^−1^ at all experimental sites except at Abala Gase where variety Dosha inoculated with strain NSFBR-15 produced the highest nodule dry weight plant^−1^. The lowest nodule dry weight plant^−1^ (106.0 mg/plant) was observed with non-inoculated + N control of variety Dosha at Hankomolicha.

Location × strain × variety interaction also had significant effect on nodule volume plant^−1^. The nodules produced varied in size while nodule volume ranged from 1.3 to 4.7 ml plant^−1^ (Table 6). Rhizobium strains inoculation significantly improved nodule volume plant^−1^ over their respective non-inoculated controls at all study locations. Rhizobium strain NSFBR-15 inoculated to faba bean variety Dosha resulted in the highest nodule volume plant^−1^ (4.7 m1/plant) at Abala Gase followed by the same strain inoculated to variety Gora (3.7 ml/plant) at Gike Atoye while the least nodule volume plant^−1^ (1.3 ml/plant) was observed with non-inoculated + N control of variety Dosha at Hankomolicha.

### *Rhizobium* strains inoculation effect on shoot dry weight

Shoot dry weight was significantly affected by location x variety x strain interaction. Rhizobium strains inoculation significantly increased shoot dry weight plant^−1^ of all faba bean varieties in all the study locations (Table 7). With the exception of EAL-110 inoculated to variety Dosha at all the study locations and NSFBR-20 inoculated to variety Moti at Haranfama, all strain x variety combinations resulted in greater shoot dry weight than non-inoculated -N control. Nitrogen fertilizer (46 kg N ha^−1^) also increased shoot dry weight with no attenuation of nodulation on non-inoculated plant.

**Table 7:** Rhizobium strain x faba bean variety interaction effects on shoot dry weight at late flowering stage.

Rhizobium strains		Varieties				Varieties		
	Locations	Moti	Dosha	Gora	Locations	Moti	Dosha	Gora
		(g plant^−1^)				(g plant^−1^)		
TAL_1035	Hankomolicha	22.0^c^	24.2^ab^	26.5^a^	Haranfama	21.1^ab^	21.5^a^	24.9^a^
NSFBR-15		25.0^b^	25.8^a^	23.3^b^		22.6^a^	19.8^bc^	24.4^ab^
HUFBR-17		21.1^c^	22.0^c^	18.6^d^		20.5^b^	19.6^bc^	17.8^c^
NSFBR-12		28.9^a^	22.6^bc^	25.1^a^		21.7^ab^	20.7^ab^	22.7^b^
EAL-110		20.6^c^	15.4^d^	18.8^d^		19.9^b^	13.7^d^	18.2^c^
NSFBR-20		16.8^e^	20.9^c^	21.3^c^		15.2^c^	20.1^b^	18.8^c^
N +		24.4^b^	24.3^ab^	25.1^a^		23.1^a^	22.5^a^	22.5^b^
N-		14.5^e^	16.8^d^	17.7^d^		17.0^c^	17.8^c^	15.8^d^
CV_a_(%)			6.2				8.0	
CV_b_(%)			7.0				8.4	
TAL_1035	Abala Gase	24.0^b^	24.4^a^	28.6^a^	Gike Atoye	23.3^a^	23.7^a^	30.7^a^
NSFBR-15		31.0^a^	25.7^a^	22.9^c^		23.9^a^	26.0^a^	28.0^a^
HUFBR-17		17.0^d^	19.0^b^	15.9^d^		20.1^b^	15.2^c^	15.9^c^
NSFBR-12		21.1^c^	23.8^a^	25.1^bc^		23.1^a^	20.9^b^	24.5^b^
EAL-110		20.2^c^	13.5^c^	15.2^d^		17.2^ cd^	18.0^c^	16.2^c^
NSFBR-20		14.9^d^	15.6^c^	17.9^d^		19.0bc	15.6^c^	14.0^ cd^
N +		25.1^b^	23.4^a^	25.8^b^		23.9^a^	24.4^a^	24.4^b^
N-		10.1^e^	14.9^c^	17.8^d^		15.3^d^	18.0^c^	10.9^d^
CV_a_(%)			12.7				10.1	
CV_b_(%)			9.5				11.6	

Mean values in the same column with different letter(s) are significantly different at p ≤ 0.05 probability level.

Faba bean variety Moti inoculated with NSFBR-15 produced the greatest shoot dry weight at Abala Gase (31.0 g plant^−1^) while variety Gora inoculated with strain TAL_1035 produced the greatest shoot dry weight at Haranfama (24.9 g plant^−1^) and Gike Atoye (30.7 g plant^−1^) (Table 7). Faba bean variety Dosha inoculated with NSFBR-15 produced shoot dry weight which was statistically at par with 46 kg N ha^−1^ treated plants at Hankomolicha, Abala Gase and Gike Atoye. Rhizobium strain NSFBR-12 with variety Moti at Hankomolicha, TAL_1035 with Moti and Gora varieties at Haranfama and Abala Gase, respectively and NSFB-15 with varieties Moti and Gora at Abala Gase and Gike Atoye, respectively resulted in significantly greater shoot dry weights than that obtained following application of 46 kg N ha^−1^ at all study locations. Rhizobium strain NSFBR-15 inoculation to varieties Moti and Gora resulted in 206.9% and 156.9% shoot dry weight increment as compared to their respective –N control treatment at Abala Gase and Gike Atoye, respectively while application of 46 kg N ha^−1^ resulted in 148.5% and 123.9% shoot dry weight increment in the same varieties at the same study locations (Table 7).

### *Rhizobium* strains inoculation effect on root dry weight

Location × strain × variety interaction had significant effect on root dry weight of faba bean. Inoculation of rhizobium strains also resulted in a significant variation in faba bean root dry weight (Table 8). Rhizobium strain NSFBR-15 inoculation to varieties Moti and Dosha resulted in the greatest RDW at Abala Gase (5.6 g plant^−1^) followed by Gike Atoye (5.5 g plant^−1^) while variety Moti inoculated with NSFBR-12 and NSFBR-17 produced the greatest RDW at Hankomolicha (7.0 g plant^−1^) and Haranfama (6.3 g plant^−1^), respectively.

**Table 8:** Rhizobium strain x faba bean variety interaction effects on root dry weight at late flowering stage.

Rhizobium strains		Varieties				Varieties		
	Locations	Moti	Dosha	Gora	Locations	Moti	Dosha	Gora
		(g plant^−1^)				(g plant^−1^)		
TAL_1035	Hankomolicha	5.4^d^	5.0^c^	6.1^ab^	Haranfama	5.7^bc^	4.8^cde^	5.4^abc^
NSFBR-15		5.7^ cd^	5.9^b^	5.0^c^		5.7^bc^	5.4^b^	4.6^d^
HUFBR-17		6.5^ab^	6.9^a^	5.6^bc^		6.3^a^	5.4^b^	5.9^a^
NSFBR-12		7.0^a^	4.8^ cd^	5.5^bc^		5.3^ cd^	4.5^de^	5.2^bc^
EAL-110		6.2^bc^	4.3^d^	5.6^bc^		6.0^ab^	4.4^e^	5.6^ab^
NSFBR-20		4.5^ef^	6.4^ab^	6.6^a^		4.4f.	6.1^a^	5.7^ab^
N +		5.1^de^	5.1^c^	5.4^bc^		5.1^de^	5.0^bcd^	4.9^ cd^
N-		4.1f.	4.7^ cd^	5.2^c^		4.7^ef^	5.2^bc^	5.0^ cd^
*CV*_*a*_*(%)*			10.0				7.5	
*CV*_*b*_*(%)*			10.0				8.3	
TAL_1035	Abala Gase	4.9^b^	4.5^ab^	4.4^ab^	Gike Atoye	4.6^b^	4.1^cde^	4.7^ab^
NSFBR-15		5.6^a^	4.9^a^	4.8^a^		5.2^a^	5.5^a^	5.2^a^
HUFBR-17		4.7^b^	4.2^b^	3.8^c^		4.9^ab^	3.6^e^	4.6^b^
NSFBR-12		4.1^c^	4.3^b^	4.2^bc^		4.4^b^	3.7^de^	4.7^ab^
EAL-110		5.4^a^	4.0^bc^	3.2^d^		4.4^b^	5.1^ab^	4.5^bc^
NSFBR-20		3.4^d^	4.3^b^	4.9^a^		4.8^ab^	4.2^ cd^	3.8^c^
N +		4.2^c^	4.0^bc^	4.5^ab^		4.3^b^	4.4^c^	4.3^bc^
N-		2.4^d^	3.6^c^	4.6^ab^		3.6^c^	4.6^bc^	3.1^d^
*CV*_*a*_*(%)*			9.5				10.8	
*CV*_*b*_*(%)*			10.5				10.4	

Mean values in the same column with different letter(s) are significantly different at p ≤ 0.05 probability level.

### Rhizobium strains inoculation effect on shoot to root ratio

Shoot to root ratios were variable and significantly (P < 0.001) affected by location × variety × strain interaction. Shoot to root ratio ranged between 3.01 and 6.63 (Table 9). The lowest shoot to root ratio (3.01) was obtained with HUFBR-17 inoculated to variety Gora at Haranfama whereas the highest ratio (6.63) was obtained by the same variety inoculated with strain TAL_1035 at Gike Atoye. Generally, inoculation with TAL_1035, NSFBR-15 and NSFBR-12 resulted in higher shoot to root ratios relative to other strains and non-inoculated -N control treatment with all varieties at all study locations.

**Table 9:** Rhizobium strain × faba bean variety interaction effects on shoot to root ratio at the study locations.

Rhizobium strain	Hankomolicha			Haranfama			Abala Gase			Gike Atoye		
	Moti	Dosha	Gora	Moti	Dosha	Gora	Moti	Dosha	Gora	Moti	Dosha	Gora
TAL_1035	4.06^bc^	4.82^a^	4.38^a^	3.70b^cd^	4.48a	4.63^b^	4.90^bcd^	5.66^a^	6.61^a^	5.02^ab^	5.86^a^	6.63^a^
NSFBR-15	4.39^b^	4.46^a^	4.67^a^	4.13^ab^	3.70^b^	5.33^a^	5.53^ab^	5.24^ab^	4.80^ cd^	4.64^bc^	4.67^b^	5.46^b^
HUFBR-17	3.24^e^	3.22^c^	3.34^b^	3.27^d^	3.67^b^	3.01^c^	3.65^e^	4.58^bc^	4.17^cde^	4.21^ cd^	4.22^bc^	3.46^c^
NSFBR-12	4.15^bc^	4.74^a^	4.59^a^	4.09^abc^	4.57^a^	4.37^b^	5.14^abc^	5.57^a^	6.07^a^	5.22^ab^	5.65^a^	5.28^b^
EAL-110	3.31^e^	3.57^bc^	3.34^b^	3.32^d^	3.13^b^	3.27^c^	3.76^e^	3.35^d^	4.94^bc^	3.89^d^	3.51^d^	3.61^c^
NSFBR-20	3.82^ cd^	3.24^bc^	3.25^b^	3.43^ cd^	3.29^b^	3.28^c^	4.41^cde^	3.66^ cd^	3.65^e^	3.94^d^	3.71^ cd^	3.74^c^
N^+^	4.81^a^	4.74^a^	4.64^a^	4.56^a^	4.47^a^	4.63^b^	6.00^a^	5.89^a^	5.80^ab^	5.62^a^	5.55^a^	5.72^b^
N^-^	3.57^de^	3.60^b^	3.43^b^	3.66^ cd^	3.46^b^	3.16^c^	4.15^de^	4.16^ cd^	3.90^de^	4.22^ cd^	3.95^ cd^	3.55^c^
*CV*_*a*_*(%)*		**5.9**			**6.0**			**10.8**			**10.7**	
*CV*_*b*_*(%)*		**6.0**			**12.5**			**13.1**			**8.3**	

Mean values in the same column with different letter(s) are significantly different at p ≤ 0.05 probability level.

The increment in shoot and root dry weight following inoculation was not proportional. Relatively, more increment was observed in shoot dry weight than root dry weight. Inoculation with NSFBR-15 and NSFBR-12 to variety Moti resulted in 206.9% and 99.3% shoot dry weight increase at Abala Gase and Hankomolicha, respectively but 133.3% and 70.7% root dry weight increase on the same variety at the same study sites over respective non-inoculated −N controls (Tables 7 and 8). Nitrogen fertilizer application also resulted in 68.3% and 148.5% increase in shoot dry weight of variety Moti while resulting in 24.4% and 41.7% increase in root dry weight at Hankomolicha and Abala Gase, respectively (Tables 7 and 8).

### Relationship between nodulation and dry matter yield

Nodulation parameters (numbers of nodules plant^−1^, nodule dry weight plant^−1^ and nodule volume plant^−1^), shoot dry weight plant^−1^, root dry weight plant^−1^, and shoot to root ratio showed varying degree of relationship (Table 10). Number of nodules plant^−1^ and nodule dry weight plant^−1^ had significant (p ≤ 0.01) positive correlation to shoot dry weight and root dry weight plant^−1^. However, nodule dry weight plant^−1^ correlated more strongly with shoot dry weight than nodule number.

**Table 10:** Correlation between traits of nodulation and dry matter yield.

	NV		NDW		SDW		RDW		SRR	
	r	R^2^	r	R^2^	r	R^2^	r	R^2^	r	R^2^
NN	0.98	0.96	0.99**	0.97	0.52**	0.27	0.51**	0.26	0.31^ ns^	0.09
NV			0.97	0.94	0.49*	0.24	0.53**	0.28	0.27^ ns^	0.07
NDW					0.63**	0.39	0.49*	0.24	0.43*	0.18

**Highly significant; * Significant; ^ns^ Not significant; *NN* nodule numbers plant^−1^, *NV* nodule volume (mg) plant^−1^, *SDW* shoot dry weight (g) plant^−1^, *RDW* root dry weight (g) plant^−1^, *SRR* shoot to root ratio.

## Discussion

The initial soil properties were examined to identify whether variability existed which could explain the occurrence and magnitude of the treatments response. Such knowledge is important to evaluate new technologies and identify the need for further research on soil fertility management options. The mean population of native faba bean nodulating rhizobia varied from 3.1 × 10^2^ to 1.7 × 10^3^ cells per gram of soil across the study locations (Table 2). Continuous cultivation may build-up rhizobia populations in the soil, resulting in increase in nodulation^39^. The higher rhizobial numbers at Abala Gase and Gike Atoye may also be due to the relatively higher soil organic carbon content (Table 2). Ulzen^40^ reported an optimal expression of symbiotic functions following an increase in organic carbon content which serve as substrate for *Bradyrhizobium* and consequently their survival. Zengeni et al.^41^ indicated that, organic manure enhanced the survival of soybean nodulating rhizobia.

Rhizobium strains inoculation under greenhouse condition resulted in significant (p ≤ 0.05) differences in number of nodules and nodule dry weight plant^−1^ (Table 3). The variations in number of nodule plant^−1^ and nodule dry weight plant^−1^ indicated that, response of tested faba bean varieties varied with rhizobium strains. This result confirms earlier reports that, faba bean gives high nodulation when suitable symbiotic partners are supplied^42^. It can thus be said that, effectiveness of faba bean-rhizobia symbiosis depends on both macro and micro-symbionts which further explains the apparent differences in compatibility between the faba bean varieties and rhizobium strains. The greenhouse experiment is useful as it is the only way to determine performance of pure strains used as inoculant under similar conditions, and thus complement the findings of field experiments. However, the validity of greenhouse experiment is limited, because it does not represent varying soil and environment conditions^43^.

The results of this study are in harmony with the finding of Farid and Navabi^44^ who reported significant interaction of host genotype with rhizobia strain and location on symbiotic traits of common bean. The establishment of introduced rhizobium strain often decreases with increase in soil resident rhizobia populations^45^. However this study revealed that, the performances of the inoculant strains were not affected by the potential competition from soil resident rhizobial strains’ population. Nodulation responses to inoculation were observed at all study locations. Similarly, Solomon et al.^46^ reported inoculation responses in soybean in the country. Inoculation ensures that the presence of the desired rhizobium strains is in close proximity to the root of the plant and thus increases the number of nodules formed by inoculant strains.

Nodulation of all non-inoculated faba bean plants in all the study locations confirmed the presence of infective soil resident rhizobia strains population across the study locations. The highest number of nodules (48.0) attained by non-inoculated + N control plant at Gike Atoye as compared to the other experimental locations could be attributed to the presence of higher population of native rhizobia at the study site (Table 2). Number of nodules formed is determined by the population size of the infective rhizobia in the soil^47^. However, non-inoculated -N faba bean bean plants produced the least average (22.0) number of nodules plant^−1^ at Abala Gase which had the second highest soil rhizobial population. Many researchers revealed that, the ability of legumes to form nodules is mainly attributed to soil and biological factors including; levels of available phosphorus, soil pH, type and vigor of legume, rhizobia population and their effectiveness^47,48^. Thus, the relatively lower soil pH coupled with low available phosphorus at Abala Gase experimental location (Table 2) might have accounted for the lower nodulation. Legumes depending on symbiotically fixed nitrogen requires high amount of phosphorus^49^ which is usually critical in low soil pH^50^.

Both non-inoculated -N and + N control treatments gave statistically equal number of nodules plant^-l^ (Table 4), an indication that nitrogen fertilizer application (46 kg N ha^−1^) did not inhibit nodulation of faba bean, although Talaat and Abdallah^51^ reported negative effects of higher soil nitrogen levels on nodulation. This can also be due to the fact that the amount of nitrogen used as control treatment (46 kg N ha^−1^) was probably not sufficient enough to affect nodulation of faba bean. On the other hand, Yoseph and Worku^52^ reported that, application of 50 kg N ha^−1^ resulted in greater number of nodules plant^−1^ and higher nodule dry weight plant^−1^ than non-fertilized control soybean plants. Similarly, Talaat and Abdallah^51^ found that, inoculation with *Rhizobium* in the presence of 50% of the recommended rate of nitrogen induced significant increase in nodulation. Variation in nodulation of faba bean due to rhizobium strains inoculation in the study locations indicated that, other edaphic factors could have modified rhizobium strain × variety interaction. Site specific plant response to rhizobial inoculants is not uncommon^53,54^ and might be attributed to differences in effectiveness of rhizobium strains and native rhizobia strain population among locations. The observed variations in nodulation due to different rhizobium strains inoculation to the same variety and the same strain to different varieties pointed out the differences in host and microsymbiont capacity for nodule formation.

Rhizobium strains inoculation resulted in significant increase in nodule dry weight of faba bean (Table 5). Voisin et al.^55^ showed that, increase in nodule dry weight was associated with enhanced symbiotic efficiency during nodule growth. Graham et al.^56^ reported that, nodule dry weight is a good indicator for symbiotic efficiency, and thus an important tool in strain evaluation. The results of this study elucidate the elevated affinity and greater competitiveness of strains TAL_1035 and NSFBR-15 over other strains tested. Nodule dry weight depends on rhizobium strain and legume variety interaction^46^. The observed differences confirm that, nodulation depends on the interactions of legume variety, the rhizobium strain and the bio-physical environment^1^.

Rhizobium strains inoculation significantly increased faba bean shoot dry weight plant^−1^ across the study locations (Table 7). The increases in shoot dry weight in the N-fertilized and/or inoculated plants as compared to non-inoculated –N control indicate that, nitrogen was a limiting factor at all study locations. On the contrary, Ulzen^40^ did not observe an increase in shoot dry weight following rhizobia inoculation on soybean but opined his finding to inefficiency of the introduced strains in nitrogen fixation. However, the results of this present study are in harmony with those obtained by Talaat and Abdallah^51^, Minalku et al.^57^, Sindhu et al.^58^ and Hemissi et al.^59^. Besides N_2_ fixed, growth hormones synthesized by rhizobium have been considered the other probable means to promote plant growth^50,61^.

Rhizobium strains, NSFBR-12 with variety Moti at Hankomolicha, TAL_1035 with varieties Moti and Gora at Haranfama and Abala Gase, respectively and NSFB-15 with varieties Moti and Gora at Abala Gase and Gike Atoye, respectively resulted in significantly greater shoot dry weight (Table 7) than 46 kg N ha^−1^ at respective study locations. The presumed higher cost of carbon to symbiotic nitrogen fixation, as compared to root mineral nitrogen absorption, did not affect shoot biomass yield. This can be explained by the good symbiotic activity induced by the rhizobium strains with respective varieties at the study locations. Efficient rhizobium strains stimulate the formation of more nodules which provides legumes with more fixed nitrogen resulting in greater biomass yield. Rhizobia inoculation increases nodulation and nitrogen fixation as a result increased plant growth^62^. The results of this study demonstrate the promising potential of rhizobium strains inoculation as successful alternatives or compliments to inorganic nitrogen fertilizer. In line with the finding of Tena et al.^63^, the results have shown that effective rhizobium strain inoculation to faba bean can reduce the need for inorganic nitrogen fertilizer to increase growth and shoot dry weight accumulation of the crop. The enhancement of plant growth and dry matter accumulation due to rhizobium strain inoculation could be attributed to the fact that, rhizobium strains augment plant growth by directly providing fixed nitrogen. The effect of rhizobium strains inoculation on plant growth might also be attributed to mobilization of insoluble nutrients that in turn increase plant nutrient uptake^64^, and/or plant growth hormone production that stimulate plant growth^51,60,61^.

Inoculation of rhizobium strains resulted in significant variation in faba bean root dry weight (Table 8). Earlier reports of inoculating legumes with rhizobium strains showed a positive effect on root dry weight^65,66^. Korir et al.^67^ claimed that, the increased activity of rhizobium strain in the rhizosphere following inoculation could increase the growth intensity of roots. However, in this study, the observed increase in shoot and root dry weights resulting from rhizobium strains inoculation was not proportional. Plants partition biomass between shoot and root to effect optimal utilization of available resources^68^. Under nutrient stress conditions, more biomass is allocated mainly to the root system resulting in an increase in the absorbing plant organ. On the other hand, plants that have adequate nitrogen supply direct biomass predominately to the shoot^68^. This explains the reason why efficient rhizobium strains like NSFBR-15 and NSFBR-12 resulted in more increase in shoot dry weight relative to root dry weight.

The variations in shoot and root dry weights among the different rhizobium strains inoculated plants revealed deviations in assimilate partitioning which led to the differences in shoot to root ratio. Inoculation with the three top rhizobium strains, NSFBR-15, TAL_1035 and NSFBR-12 led to relatively more biomass partitioning to the shoot at the expense of the root. Higher shoot to root ratio obtained with NSFBR-15, TAL_1035 and NSFBR-12 inoculation could be due to the fully functional nitrogen fixation system as a result of prolific nodulation. On the other hand, inoculation with HUFBR-17, EAL-110 and NSFBR-20 resulted in relatively lower increment in both shoot dry weight and shoot to root ratio (Tables 7 and 9). These findings suggest that, with insufficient nodulation and nitrogen fixation, faba bean plants will require an alternate source of nitrogen to maintain tissue requirement by diverting more investment to extensive root system^69^.

Rhizobium strains inoculation had significant effects on nodulation and dry matter production of faba bean (Tables 4, 5, 6, 7, 8). The study results elucidate the elevated affinity and greater competitiveness of rhizobium strains NSFBR-15, TAL_1035 and NSFBR-12 over other strains tested. Five rhizobium strains (NSFBR-15, TAL_1035, NSFBR-12, EAL-110 and NSFBR-20) resulted in higher and statistically equal nodulation under greenhouse condition (Table 3) but two (EAL-110 and NSFBR-20) of them showed lower performance under field condition. Rhizobium strains TAL- 1035, NSFBR-15 and NSFBR-12 consistently gave a significantly higher nodulation under greenhouse and field conditions (Tables 3, 4, 5, 6). The previous finding of Argaw^4^ showed that, NSFBR-15 and NSFBR-12 were the most efficient strains among 49 indigenous faba bean nodulating rhizobium strains tested under greenhouse condition. These findings confirmed that, symbiotic effectiveness of rhizobium strains is variable^4,56^. These three rhizobium strains improved significantly both nodulation and dry matter production. The result further indicated that, efficient rhizobium strain stimulates more nodules formation and consequently better plant growth. Site-specific best variety x strain combinations for nodule dry weight were variety Gora with strain TAL_1035 at Hankomolicha, Haranfama and Gike Atoye, and variety Dosha with strain NSFBR-15 at Abala Gase (Table 5) while the treatments for maximum shoot dry weight were variety Gora with strain TAL_1035 at Haranfama and Gike Atoye, and Moti with strain NSFBR-15 and NSFBR-12 at Abala Gase and Hankomolicha, respectively (Table 7). This finding indicated that, legume-rhizobia symbiotic efficiency depends on the interaction between plant genotype and rhizobium strain and the environment^70^.

Enhanced nodulation resulting from rhizobium inoculation was positively associated with shoot and root dry weight plant^−1^ (Table 10). Kawaka et al.^71^ showed that, shoot biomass depends on nodulation. Guo et al.^72^ concluded that, inoculation usually stimulates plant growth by improving nodulation and biological N_2_-fixation. In line with the finding of Gicharu et al.^73^ and Mothapo et al.^74^, the results of this study indicated that, plants which formed more nodules produced higher shoot and root dry matter. Rhizobium strains with high nodulation resulted in high shoot dry weight and shoot to root ratio of faba bean. This result suggests that, increasing nodulation through inoculation could improve plant biomass.

Correlation coefficients showed that faba bean growth in terms of shoot dry weight became more dependent on nodule dry weight. Oono and Denison^75^ suggested that, both nodule dry weight plant^−1^ and shoot dry weight plant^−1^ are good indicators for symbiotic nitrogen fixation efficiency of rhizobia strains. Increase in nodule dry weight plant^−1^ results in better nitrogen nutrition which in turn improves shoot development^76^. Kantar et al.^77^ and Ogutcu et al.^66^ opined that, nodule dry weight plant^−1^ is one of the most important indicators of effective legume-rhizobia symbiosis.

## Conclusion

Nodulation response to inoculation in the presence of high numbers of native rhizobia population is an indication that, the tested rhizobium strains are more competitive. The results have shown the need for inoculation of appropriate rhizobium strains in all the study locations. Rhizobium strains TAL_1035, NSFBR-15, and NSFBR-12 elucidated the higher affinity and greater competitiveness over the other strains tested. The efficiency of nodulation and dry matter yield depends on the compatibility between the faba bean variety and rhizobium strain and their interaction with the soil bio-physical condition.
